# Supplementary material for: The role of fragrance and self-esteem in perception of body odors and impressions of others
Source: PLoS One. 2021 Nov 15;16(11):e0258773. doi: 10.1371/journal.pone.0258773 (PMC8592444; doi:10.1371/journal.pone.0258773)
Supplement: S1 File — (PDF) [file pone.0258773.s001.pdf]

These are supplementary materials belonging to:

The role of fragrance and self-esteem in perception of body odors and impressions of others

Ilja Croijmans[1,a], Daniel Beetsma[1], Henk Aarts[1], Ilse Gortemaker[2,3,b], Monique Smeets[1,2]

1 Department of Psychology, Utrecht University, Utrecht, The Netherlands

2 UNILEVER R&D Beauty & Personal Care Science & Technology, Consumer Science, Rotterdam, The Netherlands

3 ABN AMRO Bank N.V.

[a]corresponding author, i.m.croijmans@uu.nl

[b]author Ilse Gortemaker was study manager for Unilever, and currently affiliated with 3

contents:

S1. Online supplementary materials including pre-registration document

S2. Methods and results of the sender study

S3. Stimulus video sources

S4. Additional analyses for the perceiver study

S5. Pilot study methods and results

S6. References

S1. Online supplementary materials including pre-registration document

All data collected in this study, including syntax files of the analyses, and a time-stamped document outlining the research question and hypotheses (i.e., informal pre-registration) can be found on the Open Science Framework (OSF): https://osf.io/xwtnq/

S2: Methods and results of the sender study

Measures

A combination of implicit, explicit and control measures to measure the effect of fragrance on self-esteem were used. These are described below.

Implicit measures of self-esteem. To measure state self-esteem implicitly, participants were asked to put their signature on a piece of paper, with the instruction "could you please put your signature on this piece of paper". Signature size has previously been associated with self-esteem (Zweigenhaft, 1977; Rudman, Dohn & Fairchild, 2007), in such a way that the bigger the signature size, the higher self-esteem someone has. Maximum height and width of the signatures were measured, and the diagonal in millimetres was taken as dependent variable. Signature measures were checked for inter-rater reliability by an independent researcher: Cohen's Kappa for inter-rater reliability showed excellent reliability, $\kappa = 1.00$.

The name-initial preference (NIP; Nuttin, 1985; Stieger & Voracek, 2012) effect is another way to measure implicit self-esteem. Participants rate the letters of the alphabet to what extent they like each letter on a 5-point Likert scale (ranging 1 "not at all" to 5 "very much"). Initials, i.e., the first letter of the given name and surname, are expected to be liked more relative to the other letters, and this effect covaries with self-esteem level of the participant. To calculate the NIP effect for each participant in each condition, initial ratings were taken, controlling for frequency of the letter (i.e., minimizing across participant error) as well as the relative rating of each letter by that participant (i.e., minimizing within participant error), following the I-algorithm described by LeBel and Gawronski (2009). Relative given name and surname initial liking scores were taken as dependent measure.

Explicit measure of self-esteem. Participants completed the State Self-Esteem Survey (SSES; Heatherton & Polivy, 1991). This survey is a 20 item questionnaire measuring state self-esteem. Participants rate various statements on a five-point Likert scale ranging 1 ("not at all") to 5 ("very much"). Previous validation studies show excellent internal consistency for this questionnaire (α = .92; Heatherton & Polivy, 1991). The questionnaire measures self-esteem in three dimensions:

performative self-esteem, appearance self-esteem, and social self-esteem. These three dimension scores, as well as the total SSES score, were taken as dependent measures.

Procedure for sweat collection

Following the protocol previously described by De Groot et al. (2015), sender participants shaved their armpits three days before both donation sessions, and kept an eating diary for three days. During these three days, participants were instructed to refrain from eating spicy or strong smelling foodstuffs, as well as using perfumed personal care products. Perfume free products (fragrance, shampoo, shower gel and handsoap) were provided. Participants were instructed not to wear fragrance during the day of the donor sessions. Participants were provided with a clean t-shirt to wear during the day of the first donor session, after taking their morning shower.

Upon arrival in the lab, participants were asked for their adherence to the perfume use and diet restrictions. They were then taken to a bathroom to wash their armpits with lukewarm water. In the fragrance condition, fragrance was applied by the experimenter, who, after shaking the body spray can, sprayed fragrance for two seconds evenly over the surface of the armpit, in both armpits. After this, sweat pads were weighted and fitted under their armpits and participants put on a new t-shirt first, and the other t-shirt.

Senders were seated in a soundproof cubicle, maintained at a temperature of 26° C, in front of a computer. Temperature and room humidity was recorded. They then went through three filler tasks (watching one ‘How it’s made’ video[1] on YouTube[2], playing Tetris, and completing a word search puzzle) to pass 20 minutes of time. After this, temperature and room humidity was recorded again, and participants completed the implicit and explicit self-esteem measures, and filled in the questionnaire measuring emotional state. After this, armpit sweat pads were removed, weighted, coded and vacuum sealed. Sweat pads were frozen at -20° C within 5 minutes of removal from the underarm.

[1] https://en.wikipedia.org/wiki/How_It%27s_Made
[2] www.youtube.com

Sweat pads were pooled together in white opaque plastic containers with a volume of 250ml and a 50mm diameter opening, from four different senders having the same confidence status and participating in the same fragrance condition. To maximize relevant variance, and to minimize between subject/error variance, fragrance vs. no fragrance condition stimuli were composed from the same donors, e.g., if sweat from high self-esteem donors 1, 4, 8 and 12 in the no fragrance condition was used to compose the high self-esteem, no fragrance stimulus, the sweat from these same donors in the fragrance condition was then also used to make the high self-esteem, fragrance stimulus (as in De Groot et al., 2015; 2018). A Latin square design to compose the four different types of stimuli (high self-esteem, no fragrance; high self-esteem, fragrance; low self-esteem, no fragrance; low self-esteem, fragrance) ensured full counterbalancing of which senders contributed to which perceiver stimuli.

Control measures. Several control measures were taken on the sweat donation days. Room temperature and humidity were recorded on two occasions during the donation day. Pads were weighed before and after the sweat session. These measures were analysed to check whether temperature and humidity were not deviating, but were found not to be different between conditions or between groups. Pad weights were analysed, but no significant differences between fragrance conditions or between groups were found ($p > .05$). Body spray can weight was also measured, but again no differences between the groups were found ($p > .05$).

Design & statistical analyses

The research followed a 2 by 2 mixed design. Since we include participants either scoring high or low on self-esteem, the between participant factor is Group (low vs. high self-esteem sweat donors). Participants participate in two conditions, one where fragrance was applied and one without fragrance application. The use of Fragrance (yes vs. no) is the within participant factor.

All dependent variables were subjected to mixed ANOVAs, with Group (low vs. high self-esteem sweat donors) as between participant factor, and Fragrance (no vs. yes) as within participant factor. Outliers were replaced using a Median Absolute Deviation (MAD) threshold method (Leys et al., 2013). The usual assumptions for mixed ANOVAs were checked. Since ANOVA is robust against

slight deviations from normality, only very severe deviations from normality were resolved using appropriate transformations. Pairwise comparisons to follow up ANOVA tests were Bonferroni corrected.

Results sender study

Implicit measures of self-esteem. Of the implicit measures, signature size was analysed first. There was no effect of Group on signature size, F (1, 30) = 1.35, p = .255, η_p^2 = .043, and no main effect of Fragrance, F (1, 30) = .337, p = .566, η_p^2 = .011. In addition, there was no interaction between Fragrance and Group, F (1, 30) = .510, p = .481, η_p^2 = .017.

Turning to the Name Initial Preference task, first name initial liking was analysed. There was no effect of Group on initial liking, F (1, 30) = .177, p = .677, η_p^2 = .006, no main effect of Fragrance, F (1, 30) = .101, p = .752, η_p^2 = .003, and there was no interaction between Fragrance and Group, F (1, 30) = .178, p = .676, η_p^2 = .006.

The same picture emerged when looking at the surname initial liking. There was no effect of Group on initial liking, F (1, 30) = .235, p = .632, η_p^2 = .008, no main effect of Fragrance, F (1, 30) = .100, p = .799, η_p^2 = .002, and there was no interaction between Fragrance and Group, F (1, 30) = .066, p = .799, η_p^2 = .002.

Explicit measure of self-esteem. For the total score on the SSES, there was a significant main effect of Group, F (1, 30) = 17.851, $p < .001$, η_p^2 = .373. However, the effect of Fragrance was not significant, F (1, 30) = .454, p = .506, η_p^2 = .015. These effects should be interpreted in light of a significant interaction between Fragrance and Group, F (1, 30) = 5.094, p =.031, η_p^2 = .145. Post-hoc comparisons for each group comparing Fragrance showed that self-esteem did not differ between people with low self-esteem when wearing body spray (M = 74.9, SD = 9.1) or when not wearing body spray (M = 73.1, SD = 9.4), p = .272, d = .19. However, for people with high self-esteem, self-esteem was *lower with* fragrance use (M = 83.6, SD = 8.6) than without (M = 86.9, SD = 4.9), p = .047, d = .47.

S3. Stimulus video sources

S3 table 1: list of video sections and source Youtube videos with start and end times. Videos 1-16 were used as experimental material. Videos A and B were used as training material.

vid	program	Candidate names	Youtube link	Start (seconds after start)	End (seconds after start)
**1**	First dates hotel	Juliette & Othello	https://www.youtube.com/watch?v=MtuWaWuEFeQ	134	172
**2**	First dates	Rachel & Harry	https://www.youtube.com/watch?v=2yz2l97okOg	3	39
**3**	First dates australia	Anna & David	https://www.youtube.com/watch?v=M50bSuCsJ-k	46	95
**4**	First dates	unknown	https://www.youtube.com/watch?v=6PT0BTVWK3s	113	143
**5**	First Dates hotel	Paul & Kimmy	https://www.youtube.com/watch?v=gNMdL_UCiJ4	591	626
**6**	First dates australia	Libby & Nick	https://www.youtube.com/watch?v=FbFztDqgieU	0	38
**7**	First dates australia	Bridget & Tony	https://www.youtube.com/watch?v=piNImZWZzs8	99	146
**8**	Celebrity first dates	Melinda & Chris	https://www.youtube.com/watch?v=ktiRnNyoTgE	150	177
**9**	First dates abroad	George & Amanda	https://www.youtube.com/watch?v=JqB0XzJU7pI	56	82
**10**	First dates	Ben & Leora	https://www.youtube.com/watch?v=LPWiTivhAMA	0	30
**11**	First dates	Unknown	https://www.youtube.com/watch?v=OK4Nvs-UvMI	157	194
**12**	First dates	Unknown	https://www.youtube.com/watch?v=BH70CmvRdug	0	33
**13**	First dates abroad	Tony & Simone	https://www.youtube.com/watch?v=uzOtqLKBNKo	5	34
**14**	First dates abroad	unknown	https://www.youtube.com/watch?v=T9u8C9sLRRo	118	142
**15**	Celebrity first dates	Preston & Nicole	https://www.youtube.com/watch?v=yZ4Ge0Znh5I	50	78
**16**	First dates	Anna & Liam	https://www.youtube.com/watch?v=25S6wqxdSiM	123	142
**A**	First dates	Ian & Emma	https://www.youtube.com/watch?v=6tHDNGGXOKM	0	21
**B**	First dates Australia	Eliza & Alex	https://www.youtube.com/watch?v=PW1GEqV6N9Q	59	87

S4. Additional results perceiver study

Additional psychological judgments. The answers to the statement whether participants would date the male in the video was analysed. Data for this question was severely skewed in all conditions, with a relatively normal distribution of answers in addition to many minimum answers. Usual transformations were not appropriate, since these either worsened non-normality or gave different results for different tests. Since the Friedman test is not reliable and is usually a less powered alternative to parametric ANOVAs, it was decided to do three Wilcoxon signed-rank tests, comparing both Sender types (for Deo and No deo separately), and both deo application conditions (for High and Low self-esteem donors separately).

There was no effect of Sender type, $Z_{no\ deo}\ (62) = -.960,\ p = .337;\ Z_{deo}\ (62) = -1.13,\ p = .261$. There was, however, an effect for Fragrance, but only in the low self-esteem condition: $Z_{low\ confidence}\ (62) = -2.42,\ p = .016$, and not for the high self-esteem condition, $Z_{high\ confidence}\ (62) = -.269,\ p = .788$. This may be interpreted as an interaction: men were rated as more dateable only when smelling sweat from low self-esteem men without fragrance.

Contraceptive use. Hormonal contraceptive use might affect smell sensitivity and affective judgments (Endevelt–Shapira et al., 2019; Derntl et al., 2013; Havlicek et al., 2005), although the evidence for an effect is mixed (cf. Schaefer, Iravani, Arshamian & Lundstrom, 2021). To explore these potential effects, we repeated the dependent measures self-esteem rating and attractiveness rating from the video rating task, mood and arousal change scores in all conditions, and pleasantness, and intensity ratings as well as odor discrimination results from the odor rating tasks, were analysed by means of mixed ANOVAs, with Donor and Fragrance as within participant factors as before, but with Contraceptive use (yes vs. no) as between participant factor. These analyses were exploratory in nature, are based on unequal convenience samples, and should therefore be interpreted with caution.

For the ratings of self-esteem, there was a significant main effect of Fragrance, $F(1, 60) = 4.413$, $p = .040$, $\eta_p^2 = .069$. There was no main effect of Donor, $F(1, 60) = .940$, $p = .336$, $\eta_p^2 = .015$, or a main effect of Contraceptive use, $F(1, 60) = .469$, $p = .496$, $\eta_p^2 = .008$. Turning to the interactions, there was a significant interaction between Donor and Contraceptive use, $F(1, 60) = 5.06$, $p = .028$, $\eta_p^2 = .078$. Follow up pairwise tests for each level of contraceptive use showed that women who used contraceptives rated men having more confidence when smelling sweat from *low* self-esteem men ($M = 52.8$, $SE = 1.7$) compared to when smelling sweat from *high* self-esteem men ($M = 47.6$, $SE = 1.7$), $p = .016$. This effect was not present for women who did not use hormonal contraceptives, $p = .404$. None of the other interaction effects were significant.

For attractiveness ratings, there was a significant main effect of Fragrance, $F(1, 60) = 7.949$, $p = .007$, $\eta_p^2 = .117$. There was no effect of Donor, $F(1, 60) = .181$, $p = .672$, $\eta_p^2 = .003$, and no effect of Contraceptive use, $F(1, 60) = .221$, $p = .640$, $\eta_p^2 = .004$. The main effect of Fragrance should be interpreted in light of a significant interaction between Contraceptive use and Fragrance, $F(1, 60) = 6.191$, $p = .016$, $\eta_p^2 = .094$. Pairwise tests comparing Fragrance condition per level of Contraceptive use showed that women who did not use hormonal contraceptives, when smelling stimuli containing fragrance, they rated men ($M = 37.1$, $SE = 2.9$) as more attractive than without fragrance ($M = 30.9$, $SE = 2.9$), $p = .001$. However, this effect was not present for women using hormonal contraceptives, $p = .799$. None of the other interaction effects were significant.

Turning to mood and arousal scores: for mood change from baseline scores in the different conditions, there was no main effect of Fragrance, $F(1, 60) = .001$, $p = .981$, $\eta_p^2 = .000$, no main effect of Donor, $F(1, 60) = .000$, $p = .988$, $\eta_p^2 = .000$, and no main effect of Contraceptive use, $F(1, 60) = 991$, $p = .323$, $\eta_p^2 = .016$. There was, however, a significant interaction between Donor and Contraceptive use, $F(1, 60) = 4.601$, $p = .036$, $\eta_p^2 = .071$.

These differences for the Donor groups for each level of Contraceptive use did not survive Bonferroni correction, *p*s > .100. None of the other interactions were significant. For arousal scores compared to baseline, none of the main effects or interactions were significant.

For the ratings of pleasantness, there was a main effect for Donor: $F(1, 60) = 13.232$, $p < .001$, $\eta_p^2 = .181$), indicating that sweat from men with high self-esteem was rated as being more pleasant, independent of fragrance application. There was also a strong main effect for Fragrance: $F(1, 60) = 119.848$, $p < .001$, $\eta_p^2 = .666$, indicating that sweat samples from men who wore fragrance at the time of donation were reliably rated as more pleasant, independent of the level of self-esteem of the donor. The effect of Contraceptive use was not significant, $F(1, 60) = .228$, $p = .635$, $\eta_p^2 = .004$. In addition, none of the interactions significantly affected pleasantness ratings.

For the ratings of intensity, there were significant main effects for Donor, $F(1, 60) = 6.079$, $p = .017$, $\eta_p^2 = .092$, and Fragrance, $F(1, 60) = 167.413$, $p < .001$, $\eta_p^2 = .736$. However, these effects on intensity ratings should be seen in light of a significant interaction between Donor and Fragrance, $F(1, 60)$ 11.252, $p = .001$, $\eta_p^2 = .158$. Bonferroni corrected pairwise comparisons comparing the two Donor groups for each level of Fragrance showed that without fragrance, sweat from men with low self-esteem was rated more intense ($M = 2.44$, $SD = 1.67$) than from men with high self-esteem ($M = 1.5$, $SD = 1.21$), $p = .001$. This difference was no longer significant when participants wore fragrance, ($M_{LowSE} = 4.11$, $SD_{LowSE} = 1.22$, $M_{HighSE} = 4.32$, $SD_{HighSE} = 1.10$), $p = .287$. The effect of Contraceptive use was not significant, $F(1, 60) = 1.555$, $p = .217$, $\eta_p^2 = .025$, and neither were any of the interactions.

Finally, for the discrimination task results, there were a total of 49 test instances for women not using hormonal contraceptives. Thirty-two out of these 49 test instances were correct

(65%), significantly deviating from 50%, $t(48) = 2.23$, $p = .031$. For women using hormonal contraceptives, there were 62 test instances, of which 36 were correct (58%), which was no longer significantly above chance, $t(62) = 1.28$, $p = .207$. This may suggest that hormonal contraceptives decrease a woman's ability to discriminate sweat odors from men differing on self-esteem, although the difference is very small and should be interpreted with caution.

Overall, there were effects for the use of hormonal contraceptives. These were mainly present on the discrimination task and ratings of attractiveness and confidence, i.e., had an effect on how men were judged depending on what type of odor stimulus was perceived. The effects are, however, not straightforward to interpret, and sometimes contradictive.

S5: methods and results of the pilot test to select video stimuli

The following describes the methods and procedure of the pilot-test to select videos of men to be used in the main experiment reported in Croijmans, Beetsma, Gortemaker, Aarts & Smeets:

The main objective was to select a set of 16 YouTube videos portraying men in a dating context, with low to average self-esteem relative to the entire set, as rated by women. The following questions were answered with this pilot test:

- What set of 16 videos portray men that are rated to be having low to medium self-esteem, relative to the entire set of 30 videos?
- For appearance ratings of men, is there a difference between videos with sound or without sound?

Methods

Participants

A total of 39 participants (all women) completed the survey. Participants were recruited via Amazon Mechanical Turk (MTurk), and were paid $6.67 (€6) for their participation. Participants were on average 39.8 years old (*SD* = 11.4). For the purpose of this pilot-test, participants were recruited from a general population, leading to a somewhat older sample than what was used in the actual experiment.

All participants were native speakers of English, except for two. Of these was a native speaker of Russian but spoke English on a daily basis, and one were native in Telugu. All participants were reported to be fluent in English.

Participants further answered questions about their relationship and marital status. Twenty participants reported to be married, eight were single, seven were in a relationship, three were widowed and one reported to be engaged. Participants answered a question about their sexual orientation: two participants reported to be bisexual, whereas the remaining 37 participants reported to be heterosexual. Participants reported their highest finished or current education. Fifteen reported ‘bachelor’s degree in college (4 years)’; eight reported ‘associate degree in college (2 years)’; seven reported ‘high school graduate’; five reported ‘Master’s degree’; three reported ‘some college’; and one reported to have or currently be enrolled in a doctorate degree.

Since previous work showed *own* self-esteem can influence how others are rated on their self-esteem (e.g. Brown, 1986), participants completed the Rosenberg Self-Esteem Scale (RSES; Rosenberg, 1965) to measure their self-esteem. Average score was 22.9 (SD = 5.5), which is comparable to the average score found on the RSES in large scale normative US data samples (i.e., M = 22.6, SD = 5.8; Sinclair et al., 2010).

Twenty participants were allocated to the muted condition, where videos were displayed without sound, and 19 participants were allocated to the sound condition. Participant enrolled in one or the other on MTurk based on identical task descriptions, so allocation was semi-randomized. None of the participants completed both (sound and muted) surveys.

Participants in both conditions did not differ on age, t (37) = .18, p = .856, or self-esteem, t (37) = .73, p = .471, showing the samples for both conditions were comparable.

Instruments & procedure:

Participants first gave their consent using a standardized information form. Then they answered the demographic questions (birth year, highest degree obtained, sexual orientation, relationship status, native language and spoken language).

Participants watched 30 sections of scenes from different versions of the television program *First Dates.* Scene sections were on average 29.6 seconds (*SD* = 13.4s) long. This length was selected since videos were required to be short enough to play four different videos for each condition in the main experiment, within the maximum length of each condition of 5 minutes (since it is expected that the cue effect would dissipate in around 5 minutes). Videos were always played without video controls, with set start and end times, in a frame with 480p resolution, with a width of 560 pixels and height of 315 pixels. See table S5.1 for the full list of YouTube videos.

Table S5.1: list of all videos that entered the pilot test.

video	Link	program	Candidate names	Youtube link	Start (seconds after start)	End (seconds after start)
**1**	1	First dates hotel	Juliette & Othello	https://www.youtube.com/watch?v=MtuWaWuEFeQ	134	172
**2**	2	First dates	Rachel & Harry	https://www.youtube.com/watch?v=2yz2l97okOg	3	39
**3**	3	First dates	Laura & Dan	https://www.youtube.com/watch?v=yI4e5DA0p_k	141	178
**4**	4	First dates australia	Anna & David	https://www.youtube.com/watch?v=M50bSuCsJ-k	46	95
**5**	5	First dates hotel	Ash & Rachel	https://www.youtube.com/watch?v=46rUs-IjDrU	138	176
**6**	6	First dates abroad	Mark & Bree	https://www.youtube.com/watch?v=ybrZ_5SDBN4	144	175
**7**	5	First dates hotel	Ash & Rachel	https://www.youtube.com/watch?v=46rUs-IjDrU	241	265
**8**	7	First dates	??	https://www.youtube.com/watch?v=6PT0BTVWK3s	113	143

**9**	8	First Dates	Bianca & ??	https://www.youtube.com/watch?v=D0Ov6SRsUKs	0	29
**10**	9	First Dates hotel	Paul & Kimmy	https://www.youtube.com/watch?v=gNMdL_UCiJ4	591	626
**11**	10	First dates australia	Eliza & Alex	https://www.youtube.com/watch?v=PW1GEqV6N9Q	59	87
**12**	11	First dates australia	Libby & Nick	https://www.youtube.com/watch?v=FbFztDqgieU	0	38
**13**	11	First dates australia	Libby & Nick	https://www.youtube.com/watch?v=FbFztDqgieU	176	207
**14**	12	First dates australia	Bridget & Tony	https://www.youtube.com/watch?v=piNImZWZzs8	99	146
**15**	13	Celebrity first dates	Melinda & Chris	https://www.youtube.com/watch?v=ktiRnNyoTgE	150	177
**16**	14	First dates abroad	George & Amanda	https://www.youtube.com/watch?v=JqB0XzJU7pI	56	82
**17**	15	Celebrity first dates	Brad & ?	https://www.youtube.com/watch?v=CPiB7AAEE28	0	32
**18**	15	Celebrity first dates	Brad & ?	https://www.youtube.com/watch?v=CPiB7AAEE28	78	105
**19**	16	First dates	Ben & ?	https://www.youtube.com/watch?v=LPWiTivhAMA	0	30
**20**	17	First dates	?	https://www.youtube.com/watch?v=OK4Nvs-UvMI	30	58
**21**	17	First dates	?	https://www.youtube.com/watch?v=OK4Nvs-UvMI	157	194
**22**	18	First dates	??	https://www.youtube.com/watch?v=BH70CmvRdug	0	33
**23**	19	First dates	Ian & ?	https://www.youtube.com/watch?v=6tHDNGGXOKM	0	21
**24**	20	First dates hotel	Jordan & Amy	https://www.youtube.com/watch?v=JuvQW--Hvgc	64	32
**25**	21	First dates abroad	Tony & Simone	https://www.youtube.com/watch?v=uzOtqLKBNKo	5	34
**26**	22	First dates	??	https://www.youtube.com/watch?v=-VaZKgE5mZs	0	32
**27**	23	First dates abroad	??	https://www.youtube.com/watch?v=T9u8C9sLRRo	118	142
**28**	24	First dates hotel	Tom & ??	https://www.youtube.com/watch?v=jZIrFTg7xew	0	37
**29**	25	Celebrity first dates	Preston & Nicole	https://www.youtube.com/watch?v=yZ4Ge0Znh5I	50	78
**30**	26	First dates	Anna & Liam	https://www.youtube.com/watch?v=25S6wqxdSiM	123	142

Participants then rated the male person in the video on 8 characteristics that were theoretically highly related to the construct of self-esteem. Participants were instructed they would watch short scenes of a dating show, and that they should pay particular attention to the male. Questions were phrased as follows: "*Based on your impression, how do the following characteristics apply to the male person in the video? He seems:".* Characteristics were: *self-confident; nervous; attractive; kind; outgoing; high on self-esteem; dominant; reliable*. These characteristics were answered on a 100 point slider scale, ranging 'not at all' to 'very much'. Participants then answered the question '*I would go on a date with him*' on a 100 point slider scale ranging 'completely disagree' to 'completely agree'. Participants then answered a question whether they had seen this particular section of the program before. Videos were presented in a random order. See Figure S5.1 for a screenshot of the question formatting.

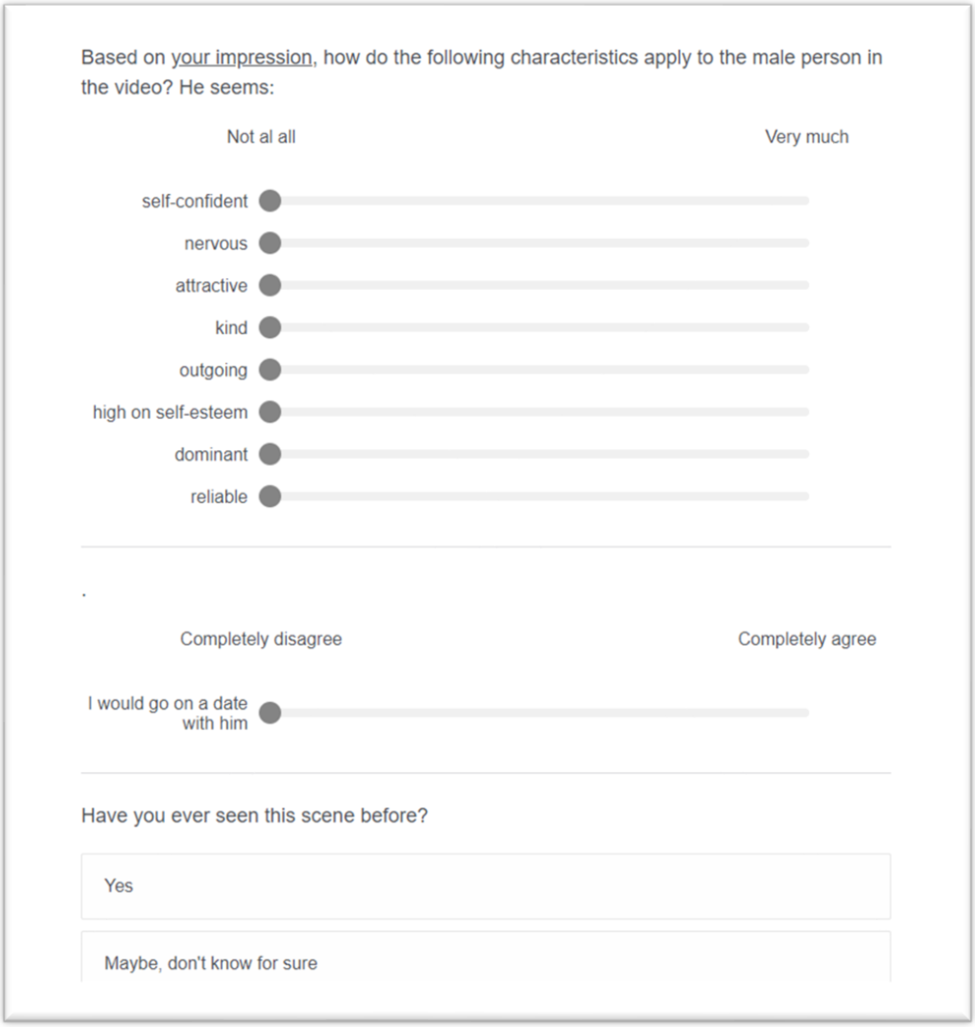

Figure S5.1: Screenshot of questions and formatting. These questions were presented in a different screen, after the video.

After watching the videos, participants answered how entertaining they found the task, rated 3 statements about the task and were given the opportunity to give any further comments about the task using an open question form.

Participant attentiveness and reliability was checked in two ways: participants answered 4 questions at random locations during the survey about specific aspects of the male characters (e.g. *what was the color of the shirt that the guy was wearing?)*, to check for their attentiveness. There was little evidence participants were inattentive to the videos (i.e., all answered at least 2 questions correctly).

A reliability analysis was also undertaken (see table S5.2), to check answer consistency between the participants, for each of the 9 questions. McDonald's omega was excellent (all >.9) for all questions, and the 'omega if item (i.e., *participant*) dropped' were all minimal (all <.05), indicating none of the participant's answer patterns markedly differed from the rest.

Table S5.2: means and standard deviations, and reliability statistics for all rating questions across all videos.

Question	Mean (SD)	Alfa	Omega
Self-confident	60.0 (10.7)	.936	.946
Nervous	51.1 (11.2)	.939	.947
Attractive	44.5 (16.7)	.945	.950
Kind	50.4 (12.6)	.945	.951
Outgoing	56.0 (13.2)	.935	.943
High on self-esteem	58.1 (12.8)	.929	.941
Dominant	51.2 (13.0)	.946	.951
Reliable	56.4 (11.8)	.943	.950
Would date	41.0 (13.1)	.917	.928

Data analysis

Means on all 9 ratings are compared between sound and muted condition to see whether sound makes a difference, using a mixed ANOVA with rating (9 levels) as within participant factor, and sound condition (2 levels) as between participant factor. Assumptions were checked and corrected for. Pairwise follow-up tests are used in case of significant interactions. In case there is a difference on the ratings of *self-confidence, high on self-esteem* and *outgoing*, the condition with the lowest ratings are selected. In case there is no difference, muted videos are selected following Roberts et al. (2009). Aim was to find videos of men scoring average on these traits, since it is expected that this leaves room for improvement using fragrance, or can be influenced by presenting sweat from men with high self-esteem.

Correlation analysis is performed to see whether the ratings correlate, and can possibly be aggregated. In addition, all ratings were correlated with participant self-esteem (measured using the Rosenberg Self-Esteem Scale) to see potential influences of rater self-esteem on the ratings of others (Brown, 1986).

After this, videos are ranked on these three variables, and 16 videos on the lower end of these ratings on these three variables (*self-confidence, high on self-esteem* and *outgoing*) are selected for the receiver task.

Note that there were 30 videos, and 39 participants. Four ratings were missing (3 in the muted, and 1 in the sound condition), amounting to DF=1166.

Results

A significant main effect of rating was not interpreted since this is out of the focus of the current research, whereas the main effect of sound was not significant, F (1, 1164) = .06, p = .810, suggesting that generally, there was no difference between the videos rated with sound or without sound.

Additionally, the mixed ANOVA showed a significant interaction between rating and sound condition, F (8, 9312) = 11.81, p = .001. Bonferroni corrected pairwise comparisons are presented in table S5.2. Importantly, for the ratings of *self-confident*, *self-esteem* and *outgoing*, sound did not matter.

Table S5.2: Bonferroni corrected pairwise comparisons for both sound conditions for the rating questions. Note: ratings in bold are the primary selection variables for the video's. Asterisks (*) mean a significant difference at α = .05.

rating	Mean (SD) Muted	Mean (SD) Sound	*P*	Cohen's *d*
Self-confident	59.7 (21.8)	60.3 (27.5)	.647	.024
Nervous	39.1 (26.3)	47.8 (33.0)	<.001*	.293
Attractive	51.5 (26.3)	50.0 (29.7)	.355	-.056
Kind	55.3 (21.7)	59.9 (25.9)	.001*	.193
Outgoing	60.0 (21.0)	59.8 (25.2)	.862	-.008
High on self-esteem	58.5 (22.3)	58.8 (27.9)	.834	.012
Dominant	47.1 (23.2)	40.1 (25.8)	<.001*	-.286
Reliable	52.8 (21.0)	53.2 (27.9)	.808	.016
Would date	40.0 (30.3)	36.2 (34.2)	.046*	-.118

Next, the correlation analysis was done (see Table S5.3). Self-confidence and self-esteem correlated highly (r = .805 > .8), indicating considerable overlap in these constructs, as expected. Self-confidence and extraversion (outgoing) also correlated highly (r = .669), yet

less than .8. In turn, extraversion and self-esteem also correlated highly (r = .737) but also less than .8. This means these constructs give additional information, and argue against the use of a single one of these to select the videos, but merit the use of an aggregate score of these three factors.

Correlations of all ratings with participant's own RSES score showed no significant strong correlations, although it seems own self-esteem negatively influences judgments of whether someone is rated friendly (*kind*) (r = -.114, p < .001) and whether someone is considered *datable* (r = -.130, p < .001).

Table S5.3: correlations between ratings and the RSES. Asterisks (*) mean a correlation is significant at α = .001. n = 1170.

rating	1	2	3	4	5	6	7	8	9
1. Self-confident									
3. Attractive	.378*	-.023							
4. Kind	.073	.276*	.503*						
5. Outgoing	.669*	-.252*	.454*	.389*					
6. High on self-esteem	.805*	-.439*	.452*	.153*	.737*				
7. Dominant	.611*	-.270*	.347*	-.027	.485*	.668*			
8. Reliable	.167*	.177*	.479*	.789*	.378*	.244*	.143*		
9. Would date	.276*	.006	.737*	.591*	.411*	.301*	.201*	.617*	
10. RSES	.019	.079	.022	-.114*	-.015	.023	.069	-.130*	-.002

Since these constructs are found to be closely related, ratings on self-confidence, extraversion (outgoing) and self-esteem were summed and averaged over participants in both conditions (muted and sound – since this was not found to be of influence), and videos were rank-ordered based on the averaged ratings (see Table S5.4). Men in videos were overall rated to have average to high self-esteem, and therefore, the 16 videos scoring around the midpoint of the 0-100 point scale (i.e., around 50, ranging 43-64) were selected. This was done since ratings on these videos are expected to be influenced most by contextual factors such as a chemical cue in sweat or perfume, as they leave most room for improvement by these factors. Two videos were from the same First Dates episode, starring the same man. To have no

overlap in the videos, the video with that man scoring highest on the measures was replaced with the video ranked next lowest (i.e., ranked 17).

Participants answered four multiple choice questions and one open question about the task after the main procedure. These were more qualitatively interpreted to get a sense of the participant's experience of the procedure. Participants did not find the muted condition (M = 66.6, SD = 27.1) less entertaining than the sound condition (M = 75.9, SD = 26.8), p = .287. Participants found both videos in both conditions to be long enough to form an impression (i.e., *'The videos were long enough to get a good impression';* M_{sound} = 63.7, SD_{sound} = 32.6; M_{muted} = 60.4, SD_{muted} = 30.6, p = .743). Participants also rated videos to feel equally 'long' and on the whole indicated videos were not too long (i.e., '*The videos were too long*', M_{sound} = 7.8, SD_{sound} = 12.0; M_{muted} = 14.1, SD_{muted} = 17.0, p = .193). Finally, participants in the muted condition indicated they would have needed the sound more than in the sound condition, although ratings were relatively high in both conditions (M_{sound} = 96.7, SD_{sound} = 5.6; M_{muted} = 60.6, SD_{muted} = 36.3, p <.001). This latter rating may be taken as an indication that the muted videos contain somewhat more ambiguity, and that participants focused on other contextual cues to base their rating on, which is both desired for a task in which the odor is supposed to give context. The final open question suggested that almost all women found the task interesting and entertaining to do, and further commented to find the show entertaining.

Table S5.4: ranked ratings per video section. Videos printed in bold are scenes from the same Youtube episode. Video sections marked grey are selected for the receiver task.

Rank	Video section	Youtube video	Mean rating muted, *n* = 20 (*SD*)	Mean rating with sound, *n* = 19 (*SD*)	Total mean rating, *n* =39 (SD)
1	23	19	34.48 (17.29)	31.63 (18.26)	33.09 (17.59)
2	4	4	42.52 (15.13)	46.53 (23.65)	44.47 (19.58)
3	21	**17**	46.50 (16.27)	42.68 (20.76)	44.64 (18.44)
4	22	18	46.93 (19.49)	40.26 (21.72)	43.68 (20.61)
5	27	23	49.38 (18.31)	46.77 (20.61)	48.11 (19.25)
6	19	16	51.03 (15.52)	55.98 (21.39)	53.44 (18.53)
7	2	2	51.43 (17.96)	55.79 (20.79)	53.56 (19.26)
8	25	21	51.85 (14.41)	60.67 (21.82)	56.15 (18.69)
9	29	25	52.63 (16.34)	69.61 (22.34)	60.91 (21.07)
10	10	9	52.95 (14.58)	48.49 (30.31)	50.78 (23.38)
11	8	7	56.18 (21.58)	55.07 (28.03)	55.64 (24.60)
12	16	14	57.32 (16.57)	48.72 (25.96)	53.25 (21.67)
13	1	1	59.12 (20.95)	67.37 (23.88)	63.14 (22.52)
14	14	12	59.45 (14.91)	56.46 (15.29)	57.99 (14.97)
15	20	**17**	59.72 (13.79)	46.95 (23.61)	53.50 (20.02)
16	15	13	61.18 (19.93)	62.63 (17.43)	61.89 (18.52)
17	12	**11**	62.98 (18.20)	65.23 (19.61)	64.08 (18.68)
18	30	26	64.23 (14.94)	84.51 (14.97)	74.11 (17.98)
19	3	3	64.42 (15.32)	59.53 (21.48)	61.97 (18.57)
20	28	24	65.77 (19.84)	69.98 (18.48)	67.82 (19.05)
21	26	22	66.23 (18.50)	48.63 (18.07)	57.66 (20.13)
22	11	10	66.83 (15.55)	86.26 (12.28)	76.30 (17.00)
23	9	8	67.25 (16.88)	69.33 (23.40)	68.26 (20.07)
24	5	**5**	67.45 (17.19)	62.47 (27.60)	65.03 (22.69)
25	24	20	67.53 (18.92)	67.54 (17.77)	67.54 (18.12)
26	6	6	67.60 (18.08)	70.86 (18.28)	69.19 (18.01)
27	7	**5**	69.88 (17.97)	73.51 (19.41)	71.65 (18.53)
28	18	**15**	70.30 (15.34)	76.44 (20.23)	73.29 (17.92)
29	17	**15**	73.23 (14.96)	67.25 (27.41)	70.32 (21.84)
30	13	**11**	73.67 (18.80)	51.35 (26.58)	62.79 (25.28)

Conclusions & recommendations for main study

The results show there is enough variation in the ratings of the men in the video material to be useable. Since the recommended 16 videos leave room for improvement, since they vary around the scale midpoint, it is recommended to use these 16 videos in the receiver task.

There was no difference in ratings of self-confidence, self-esteem, extraversion, and, also of interest, attractiveness, between the sound and muted condition. Muted videos offer the most ambiguous context, leaving room for the influence of smell. In addition, previous work

(Dalton et al., 2013; Roberts et al., 2009) also used muted videos in similar tasks. Therefore, it is recommended to use the muted videos.

It is also recommended to use two videos for training, to make participants familiar with the task and the different questions. To implicitly anchor participants, it is recommended to use the two unique videos that score the highest and lowest on this ranking, i.e., video section 19 and 17.

S6. References

Brown, J.D. (1986). Evaluations of Self and Others: Self-Enhancement Biases in Social Judgments. *Social Cognition*: 4(4), 353-376. https://doi.org/10.1521/soco.1986.4.4.353

Craig Roberts, S., Little, A. C., Lyndon, A., Roberts, J., Havlicek, J., & Wright, R. L. (2009). Manipulation of body odour alters men's self-confidence and judgements of their visual attractiveness by women. *International Journal of Cosmetic Science*, *31*(1), 47-54.

Dalton, P., Mauté, C., Jaén, C., & Wilson, T. (2013). Chemosignals of stress influence social judgments. *PloS one*, *8*(10), e77144.

de Groot, J. H., Smeets, M. A., Rowson, M. J., Bulsing, P. J., Blonk, C. G., Wilkinson, J. E., & Semin, G. R. (2015). A sniff of happiness. *Psychological science*, *26*(6), 684-700.

de Groot, J. H., van Houtum, L. A., Gortemaker, I., Ye, Y., Chen, W., Zhou, W., & Smeets, M. A. (2018). Beyond the west: Chemosignaling of emotions transcends ethno-cultural boundaries. *Psychoneuroendocrinology*, *98*, 177-185.

Derntl, B., Hack, R. L., Kryspin-Exner, I., & Habel, U. (2013). Association of menstrual cycle phase with the core components of empathy. *Hormones and behavior*, *63*(1), 97-104.

Endevelt–Shapira, Y., Pinchover, L., Perl, O., Bar, E., Avin, A., & Sobel, N. (2019). Women Have Reduced Ability to Discriminate Body Odors During the Withdrawal Period of Oral Contraception. *Chemosensory Perception*, 1-9.

Havlicek, J., Roberts, S. C., & Flegr, J. (2005). Women's preference for dominant male odour: effects of menstrual cycle and relationship status. *Biology letters*, *1*(3), 256-259.

Heatherton, T. F., & Polivy, J. (1991). Development and validation of a scale for measuring state self-esteem. *Journal of Personality and Social psychology*, *60*(6), 895.

LeBel, E. P., & Gawronski, B. (2009). How to find what's in a name: Scrutinizing the optimality of five scoring algorithms for the name-letter task. *European Journal of Personality*, *23*(2), 85-106.

Leys, C., Ley, C., Klein, O., Bernard, P., & Licata, L. (2013). Detecting outliers: Do not use standard deviation around the mean, use absolute deviation around the median. *Journal of experimental social psychology*, *49*(4), 764-766.

Nuttin Jr, J. M. (1985). Narcissism beyond Gestalt and awareness: The name letter effect. *European Journal of Social Psychology*, *15*(3), 353-361.

Rosenberg, M. (1965). Rosenberg self-esteem scale (RSE). *Acceptance and commitment therapy. Measures package*, *61*, 52.

Rudman, L. A., Dohn, M. C., & Fairchild, K. (2007). Implicit self-esteem compensation: automatic threat defense. *Journal of personality and social psychology*, *93*(5), 798.

Schaefer, M., Iravani, B., Arshamian, A., & Lundström, J. N. (2021). No Evidence That Hormonal Contraceptives Affect Chemosensory Perception. *i-Perception*, *12*(1), 2041669520983339.

Sinclair, S. J., Blais, M. A., Gansler, D. A., Sandberg, E., Bistis, K., & LoCicero, A. (2010). Psychometric properties of the Rosenberg Self-Esteem Scale: Overall and across demographic groups living within the United States. *Evaluation & the health professions*, *33*(1), 56-80.

Stieger, S., Voracek, M., & Formann, A. K. (2012). How to administer the initial preference task. *European Journal of Personality*, *26*(1), 63-78.

Zweigenhaft, R. L. (1977). The empirical study of signature size. *Social Behavior and Personality: an international journal*, *5*(1), 177-185.
