# Supplementary material for: The role of fragrance and self-esteem in perception of body odors and impressions of others
Source: PLoS One. 2021 Nov 15;16(11):e0258773. doi: 10.1371/journal.pone.0258773 (PMC8592444; doi:10.1371/journal.pone.0258773)
Supplement: Attachment — Submitted filename: Response to reviewers 20210413.docx [file pone.0258773.s002.docx]

Dear Editor and Reviewers,

Thank you for the opportunity to revise our manuscript following your comments. The reviews allowed us to improve the manuscript on several points following the comments of the reviewers: the order of the introduction was changed, we improved the clarity of the methods section, and have added a few points in the discussion. In addition, we report on an additional analysis in the supplementary materials, and have added many more materials to this file. In addition, we have updated the manuscript to follow the APA guidelines regarding gendered language (i.e., have changed ‘males’ and ‘men’ to ‘male participants’, etc.). We have addressed all comments and suggestions below in a point-by-point manner, with the reviewer’s comments in *italics*, and our response in normal typeface.

Following your suggestions regarding the funding statement, we have updated the funding statement & author contributions section.

*Reviewer #1: In this study, the authors explored whether body odors can communicate information about one’s self-esteem, and the role of applied fragrance in this relationship. Even though high self-esteem sweat was rated more pleasant and less intense than low self-esteem sweat, there was no difference in perceived self-esteem and attractiveness of males in videos, hence explicit differences in body odor did not transfer to judgments of related person characteristics. When fragrance was added to the body odor, males were rated as having higher self-esteem and being more attractive.
The research question is well explained and related to the previous literature. Experiments have been conducted rigorously, with appropriate controls and sample sizes. I particularly appreciated the use of Bayesian analyses in support of frequentist analyses. The discussion is properly supported by the results. I have just few points that need to be addressed.*

We would like to thank the reviewer for their positive reading of our paper and their suggestions for improvement of the manuscript.

*- I would suggest to change the title in order to give a sense of the findings. Right now, it sounds like body odors can communicate self-esteem*

We agree the title does not cover the article fully, and have changed it to:

“The role of fragrance and self-esteem in perception of body odors and impressions of others”

*- At the end of the introduction, authors should present their hypotheses*

We have changed the final paragraph so that these explicitly state our hypotheses (line 124-141):

Our hypotheses and expectations were stated as follows (see https://osf.io/s9xpq/ for pre-registration document):

1. If self-esteem is cued in body odor, exposure to sweat from men varying in self-confidence will modulate social judgments, even of other men, related to self-esteem in women: exposure to sweat from low self-esteem men will cause lower perceived self-esteem compared to exposure to sweat from high self-esteem men.
2. If fragrance masks low self-esteem, sweat obtained from men with low self-esteem treated with fragrance will lead to higher perceived self-esteem in women.

*- In the perceiver study, participants are women rating their impression of men. Did the authors control for the use of hormonal contraception and menstrual cycle? Previous literature has shown that these can affect the perception of body odors as well as social preferences so it might particularly relevant for the present study (**Endevelt–Shapira et al., 2020; Derntl et al., 2013; Havlicek et al., 2005). If the authors collect this information it might be interested to see if the results are affected by these aspects.*

Being aware of this literature, we did collect data on the use of hormonal contraceptive use by receivers. We did run these analyses, with contraceptive use as a between participant factor and use of Deodorant and Self-Esteem level of the donor as within participant factors. Since it was not part of our hypotheses, we did not report the results in the main manuscript, but instead report these in the Supplementary materials.

The results reflected effects of the use of hormonal contraceptives and the rated impression the males in videos. Women who used hormonal contraceptives rated men in videos as more confident when smelling sweat from low self-esteem men*,* (p = .028) This effect was not present for women who did not use hormonal contraceptives (*p* = .404). For attractiveness, women who did not use hormonal contraceptives rated males in videos as more attractive with deodorant present compared to without deodorant (*p* = .001). This effect was not present in women who used hormonal contraceptives (*p* = .799).

We feel these analyses do not undermine the main conclusions of the manuscript related to our primary hypotheses. In addition, the groups were unbalanced, and these analyses are post-hoc and exploratory in nature, so we have reported these in the supplementary materials (S4). However, hypotheses can be derived from these findings to be tested in a study designed to do so.

We also collected data on receiver’s menstrual cycle by means of self-report (i.e., days since last menstruation and average duration of a typical menstrual cycle), but have not analysed this data. Menstrual cycles are somewhat unpredictable with large intra and inter-individual differences (Wood, Koch & Mansfield, 2007; Guo, Manatunga, Chen & Markus, 2006), and thus self-report is a highly unreliable measure, compared to for example serum hormonal concentration measurement or saliva luteal hormone concentration (e.g., Bullivant et al., 2003).

*- The procedure is a bit complicated, maybe a figure would help the reader to understand the different steps, odor stimuli and tasks.*

We have added a flowchart outlining our procedure, see fig. 1 and 2 on lines 167 and 192 of the manuscript.

*Reviewer #2: Thank you for a possibility to review this submission. I believe that the topic is worth investigation and I found the study interesting and methodologically excellent. However, I also believe that the manuscript needs some work before it is published. My comments are mostly related to the writing style, missing details of the study and problems related to references (missing or erroneously applied). For example, the literature review in the introduction is good, but this section needs a bit of a reorganization and some shortening. One major change I would suggest is adding an analysis comparing baseline self-esteem assessments of the videos with the ratings performed during odor stimulation. Please find my detailed comments below.*

We would like to thank the reviewer for their highly positive take on our study and methods, and thank them for their highly constructive feedback.

*Specific comments:
Abstract -
Please specify that the fragrance you mention in the abstract was a fragranced body odor.*

*„This suggests body odors associated with self-esteem have distinct perceivable features, but only fragrance affects the psychological impression someone makes.” - please rephrase, this sentence is not clear.*

We would like to thank the reviewer for pointing out this was not clear in the abstract, and have taken both comments in consideration when revising the abstract (lines 20-26)

When the body odor was fragranced using a fragranced body spray, males were rated as having higher self-esteem and being more attractive. The finding that body odors from males differing in self-esteem are rated differently and can be discriminated suggests self-esteem has distinct perceivable olfactory features, but the remaining findings imply that only fragrance affect the psychological impression someone makes. These findings are discussed in the context of the role of body odor and fragrance in human perception and social communication. *Introduction –*

*Please reorganize the introduction. The first paragraph (currently lines 29-38) should be placed after the paragraph on self-esteem (currently ending at line 63).*

We agree that order makes more sense, and have reorganized the introduction following these suggestions.

*Paragraph on signal vs. cue (l. 64-76) is overly complicated and not entirely necessary. Please remove it (you can shorten the content and add it to the next paragraph).*

We agree this is rather technical and probably unnecessary, and have removed half of this paragraph and integrated the other half in the next paragraph (lines 55-71):

Recent research on the sense of smell shows that humans are sensitive to, and rely on, body odors for social communication. This so-called *social* *chemosignaling* (cf. Wyatt, 2015), allows people, for example, not only to spot the tell-tale signs of inflammation from looking at a picture of an individual, but also to detect illness from the smell that person secretes (Olsson et al., 2014). In a similar vein, people can infer stable trait characteristics such as gender and relational status (e.g., Mahmut & Stevenson, 2019; Penn et al., 2007), and personality traits such as dominance and extraversion from body odors (Sorokowska, Sorokowski, & Szmajke, 2012). In addition, body odors enable people to gauge more temporary emotional states, such as fear (e.g., Kamiloğlu, Smeets, de Groot, & Semin, 2018; Zhou & Chen, 2009), disgust (Kamiloğlu et al., 2018), and anxiety (Albrecht et al., 2011). Body odors may act as a signal, sent as a means for communication on purpose (e.g., when applying a perfume to make a particular impression when on a date), or as a cue that is understood in a particular way up by the perceiver, but not purposefully sent, (i.e., when someone gets a certain impression from someone else, by their particular body odor that is not directly manipulated; cf. Clark, 2018). Here, we investigate whether differences in self-esteem may lead to specific cues in body odors.

*The Sorokowska et al reference in line 77, Croy et al reference in line 86 and Roberts et al reference in line 123 are not well-matched to the content. Sorokowska et al study should be discussed in the paragraph on fragrances.*

We thank the reviewer for thinking with us here, and agree that the Sorokowska et al. (2016) better matches the content of the latter paragraph and moved it to line 101.

We believe that Croy, Frackowiak, Hummel & Sorokowska (2017) does match the content of line 82-83 since this publication discusses (among other things) the role of the HPA axis in changes of body odor during development. We have added a reference here to Chopra, Baur & Hummel (2008) that also deals with this topic, and have moved the reference closer to the statement on the HPA axis:

Another alternative route in which self-esteem *coincides* with a different body odor is that a different response of the HPA axis in potentially stressful situations affects the composition of sweat (e.g., Croy, Frackowiak, Hummel & Sorokowska, 2017; Chopra, Baur, & Hummel, 2008), and thus, ones’ body odor.

We furthermore believe that we accurately describe the experiment from Roberts et al., (2009) before the respective reference:

Interestingly, fragrances people wear can also have an effect on their *own* behavior: when males wore perfume during recording of videos compared to a control condition, female observers rated these males on videos as more dominant, confident, and attractive, traits closely related to self-esteem (Fontaine, Scherer, Roesch, & Ellsworth, 2007), even when women were unable to smell the males’ body odors (Craig Roberts et al., 2009).

*In the summary of the introduction „ In the present study, we examined for the first time whether self-esteem is a signal that can be inferred from body odor, and second, whether such signal changes social impressions.” - I believe you suggested that you referred to odors as cues?*

We would like to thank the reviewer for pointing out this inconsistency in the manuscript. We have changed ‘signal’ to ‘cue’ wherever appropriate.  *Methods:
„This sample size of 32 sender participants was based on what was necessary for the perceiver study: 2 groups of 16 sender participants would provide enough sweat pads for 64 perceiver participants.” - please explain the rationale behind the selection of this sender sample size - how did you calculate that?*

The 16 senders in both self-esteem conditions provide 2x8 pad pieces per fragrance condition, totaling 256 . Every stimulus is composed of 4 different pad pieces. 256 divided by 4 is 64. The power calculation of the perceiver study showed we needed to recruit female 46 perceivers (lines 185-196), but we aimed to recruit 66 of which 62 actually participated (line 185). To account for dropouts or pads we could not use, we recruited 16 participants per self-esteem group.

We have clarified this in the manuscript (lines 160-169):

This sample size of 32 sender participants was based on what was necessary for the perceiver study: per fragrance condition, a sender participant provided 2 sweat pads, one for each arm, that were cut into 8 pieces. Stimuli were composed of 4 different pieces from 4 donor participants in the same condition and group. Thus, 16 sender participants in each group would provide enough sweat pads for ( $\frac{2*8}{4}*16=)$ 64 perceiver participants.

*Please add a few words about the RSES in this section.*

We have added the following explanation of the RSES to the methods section in line 148-158:

“The RSES is a short questionnaire with 5 positive phrased and 5 negative phrased statements on self-esteem, answered using a 4-point agreement scale (ranging ‘strongly agree’ – ‘strongly disagree’). An example item is “On the whole, I am satisfied with myself”. The Dutch version of the RSES has attested good validity and internal consistency (Franck et al., 2008).”

*„During the sender study, additional measures of state self-esteem were collected.” - do you mean that the body odor donors were subject to one more measurement?*

Yes, that is what we mean. Donor participants also completed the State Self-Esteem Survey (Heatherton & Polivy, 1991), a signature size task (Zweigenhaft, 1977), and a name-initial preference task (Nuttin, 1985) in both body spray conditions. In the supplementary materials S2, we report on these additional measures of implicit and explicit self-esteem, and report the analyses done on with these measures in relation to using fragrance, under the heading ‘Results Sender Study’. We have reported these analyses in the supplementary materials since we believe this is beyond the scope of the main hypotheses of the study. We have clarified this in lines 179-184:

During the sender study, additional measures of state self-esteem were collected during both fragrance conditions: the State Self-Esteem Scale (Heatherton & Polivy, 1991), a measure of signature size (Zweigenhaft, 1977; Rawal, Harmer, Park & O’Sullivan, 2014), and the Name Initial Preference Task (Nuttin, 1985; Stieger, Voracek, & Formann, 2012; Stieger & LeBel, 2011). We did not find an effect of fragranced body spray on self-esteem in these measures. These are reported in full in the supplementary materials S2.

*„Participants were screened” - screened for what?*

We screened donor participants for the following things:

- Age between 18 and 35
- No concurrent psychological disorders
- Healthy
- Participants completed the RSES before they were invited to participate in the donor study

We screened perceiver participants for the following things:

- Not pregnant
- Heterosexual
- Age between 18 and 35
- Normal olfactory functioning as indicated by self-report
- Otherwise healthy

We have reported this intertwined in the paragraphs on the sender and perceiver participants (lines 140-154; 180-203).

*„Five participants scored one out of three correctly identified smells” - were they eliminated from the study?*

According to Hummel, Pfetzing & Lotsch (2010), the q-Sticks test detects olfactory dysfunction in a sensitive way when participants correctly identify 0 out of three smells. Based on this, we decided not to exclude participants, since every participant scored at least 1 correct. The exclusion criteria thus was 0 smells correctly identified. In addition, every participant self-reported normal olfactory functioning. We have clarified this in lines 201-208:

“Hummel, Pfetzing and Lötsch (2010) report that when using 3 sniffin’ sticks, a score of zero correctly identified smells sensitively indicates olfactory dysfunction.”

*Please describe the videos in the „stimuli” section. Did the videos present the odor senders?*

No, the video’s did not present odor senders, and we have clarified this by using the term ‘contestants’. Since we felt the videos were part of the psychological judgment task, we have added much more information on the video stimuli under the heading **Psychological Judgments**. We would also like to draw attention to our supplementary materials S3 and S4, that contains a report on the pilot study we undertook to select videos, and a full list of all videos including links to the YouTube materials used.

*You should also provide some references in the body odor section, showing that the procedure you used was valid and well-established.*

We would like to thank the reviewer for making us aware of this oversight. The final sentences in our introduction, describing the sender-perceiver paradigm, is now referenced (lines 137-141):

These hypotheses were studied using a state-of-the-field sender-perceiver paradigm (e.g., De Groot et al., 2018; Kamiloglu et al., 2018; Smeets et al., 2020; Sorokowska, Sorokowski & Havlicek, 2016), where first, sweat samples were collected from two groups of males, differing in self-esteem and either wearing fragrance or not, and second, where these samples were presented to a group of female perceivers.

And we have added references to the body odor section (line 211):

Body odors were collected following previously established sweat collection protocols (Kamiloglu et al., 2018; De Groot et al., 2018).

*Affective judgments are rather psychological judgments - please come up with a more exact name for this section.*

Following the suggestion, we have changed the section name to ‘psychological judgments’

*“Affective judgments. A task was designed to elicit affective judgements in a setting in which self-esteem and olfaction plays a role, i.e., interpersonal romantic interaction.” - this is completely unclear.*

We have greatly expanded and clarified on the task used to elicit the judgments from perceivers (lines 238-265):

**Psychological judgments**. A task was designed to elicit affective psychological judgements in a setting in which self-esteem and olfaction plays a role, i.e., interpersonal romantic interaction. Short videos showing interactions between a male and a female going on a first date were used as relevant test material, as self-esteem and body odor may modulate these interactions in an implicit manner. eighteen videos from a television dating show were selected, based on a pilot study (see the Appendix table for a list of the videos used, and Supplementary materials S4 for a report on the pilot study). Videos were selected portraying male contestants that were in the middle ranges of rated self-esteem, so that effect of sweat odor could or fragrance stimuli in either direction could be assessed. The videos were presented as embedded video with a width of 560 pixels and height of 315 pixels. All videos were played without sound (see Supplementary materials S4 for a discussion on this), and lasted for approximately 30 seconds.

After watching each video, participants were asked to rate the male on three characteristics (‘given your impression of the man, how would you rate the man in the video on:’, or ‘Afgaande op jouw indruk van de man, in hoeverre vond je de man in het filmpje:’, in Dutch), i.e., self-esteem (‘zelfverzekerd’ in Dutch), attractiveness (‘aantrekkelijk’), and extraversion (‘extravert’ in Dutch), using a 0-100 visual analog scale ranging “not at all” to “very much”.

As a brief training, before the odor conditions, participants watched two videos with male contestants that in the pilot-test were rated respectively higher and lower than the stimulus materials used during the conditions, to implicitly anchor participants in their choice and to make them familiar with the task. These videos are marked A and B in the Appendix table. Then, the actual experiment started. Four trials with different scenes were used for each of the four smell conditions, making a total of 18 trials for the entire procedure (training and experiment – see Appendix). Scenes were presented in counter-balanced fashion using a Latin square randomized design, ensuring that every participant watched a different set of videos in each sweat type condition. The scores on each of the three questions were averaged over the four different videos in the same condition and taken as dependent measures.

*Mood and arousal - can you provide more details on the original scale?*

We have added more information on this scale (lines 266-279):

**Mood and arousal.** Mood and arousal was measured using six semantic differentiation scale questions after the statement “I would like to know how you feel at this moment. Could you indicate below whether how you feel matches with:” (“graag zou ik willen weten hoe je je op dit moment voelt. Kun je hieronder aangeven in hoeverre je gevoel overeenkomt met:”), with the pairs: good-bad (“goed-slecht”); sad-happy (“bedroefd-vrolijk”); pleased-displeased (“tevreden-ontevreden”); calm-excited (“kalm-opgewonden”); tired-energetic (“vermoeid-energiek”); sedate-aroused (“rustig-opgewekt”). People indicated their response by selecting their answer on a ten-point scale. The first three questions reflected mood, and the latter three questions measure arousal. These questions were taken from Aarts and Dijksterhuis (2003), who based it on the Affect-Arousal Scale from Salovey and Birnbaum (1989). The two sets (reflecting mood and arousal respectively) of three questions were averaged per condition/baseline, scores for the four conditions were subtracted from a baseline measure taken before onset of the first odor trial to reflect change from baseline.

*L. 240 - how were the odors presented?*

We have added a clarifying sentence to this section (line 292-297):

“They were then instructed to knock on the door to alert the experimenter, who would place the first odor stimulus. The four stimulus jars for each participant were thawed 30 minutes before the start of the experiment. Just before the onset of the condition at hand, the lid was removed, and the 250ml PE container for that condition was fixed in a clamp attached to the chin-rest at a fixed distance of 2 cm from their nose.”

*Please provide a reference for the median-absolute-deviation (MAD) outlier procedure.*

We have added the reference to Leys, Ley et al., 2013 on line 323. *Results
“distributions looked close to normal” - this is oddly phrased. Maybe you can refer to skeweness and kurtosis here?*

We now refer to skewness and kurtosis in line 368-373.

However, no clear pattern of abnormality emerged for the four conditions, making a single transformation that corrected the different shifts from normality difficult, and distributions looked *close* to normal. Relative normality was further confirmed by skewness values, that were between [-.721; 1.064] for all ratings, and by kurtosis ratings, that were between [-.1.209; .265], and were thus all between the cutoff values for normality of -1.96 and +1.96 (Kim, 2013).

*I understood from the methods that you have the baseline ratings of perceived self-esteem of the males in the videos? Please provide an additional analysis, comparing these ratings to the ratings performed in a presence of an odor.*

To clarify: we do not have baseline ratings of perceived self-esteem of the men in the videos, as rated by the perceiver participants without smelling any of the stimuli. We have done a pilot-test to select videos for the experiment. Those ratings were collected in a different sample collected via Amazon MTurk. We do not see how we could somehow use those ratings in a meaningful analysis.

To clarify further what we did exactly, we have included a report on the pilot study and how video stimuli were selected in Supplementary Materials S5. We have further clarified that perceiver participants watched videos of men that were different from the men sweat was collected from, and that the videos was randomized. The presentation of the videos in the perceiver experiment was randomized using a Latin square procedure, leading to a different set of videos for each odor condition for each participant. This procedure mitigates any potential differences between the perceived self-esteem of the males in the videos, and allowing to trace any difference between the conditions due to the odor stimuli alone.

We have clarified that we do not have baseline measures in line (287-297):

They first did two practice trials of the video rating task with videos portraying a dating situation different from the videos used in the odor conditions, including the mood-arousal questions, to establish their mood-arousal baseline.

We have also revised the section on the psychological judgment task to clarify this further (lines starting 256).

*Discussion
“ The observation that low self-esteem body odor was perceived as less pleasant and more intense, did not lead to negative social impressions, suggests that feeling negative about oneself, or positive, for that matter, does not necessarily produce a smell that serves a basic social chemosignaling function.” - please rephrase, this is very difficult to follow.*

We agree that this sentence is unnecessarily difficult to follow, and have rephrased it as follows (line 472-489):

“Although low self-esteem body odor was perceived as less pleasant and more intense, this did not translate into negative social impressions. This suggests that feeling negative or positive about oneself, as indicated by self-reported self-esteem, does not necessarily translate into a social cue that affects others.”

*“One possible explanation is that the receiver participants were implicitly distracted by a mismatch between the pooled sweat stimuli, and the males in the videos they were rating.” - I do not understand this rationale.*

We have clarified this explanation for our results as follows (line 500-505):

“One possible explanation is that the receiver participants were implicitly distracted by a mismatch between the pooled sweat stimuli obtained from the male senders, and the contestants in the videos they were rating. In the current experiment, the videos portrayed contestants different from the body odor senders. The results may have been different if the videos of men portrayed the participants who donated the body odors, since that would align appearance with odor.”

*“while explicit measures (such as attractiveness ratings) usually do not show differences.” - please provide some references here - I do not find this sentence convincing.*

We have added more explanation of this point and added a reference in support (lines 513-524):

“Another possible explanation for the absence of self-esteem body odor effect on social perception and behavior concerns the nature of our test setting. Previous research on social chemosignaling (e.g., Albrecht et al., 2011; Kamiloğlu et al., 2018) mostly established effects of body odors on implicit measures of social evaluation or communication (such as facial electromyography or speeded classification of emotional facial expressions). In this case females provided explicit ratings of attractiveness and self-confidence. It is possible that social chemosignalling manifests at an implicit level of information processing as this rarely reaches consciousness (Haviland-Jones, McGuire & Wilson, 2016). Accordingly, although differences between high and low self-esteem were perceivable on explicit evaluative responses to the body odor itself (i.e. intensity, pleasantness and ability to discriminate), these difference did not affect the participants’ s social judgments in a social setting.”

*“Our findings do reveal fragranced body odor affects females’ judgments of self-esteem and attractiveness of males.” - please rephrase.*

We have rephrased this sentence (line 525) as:

“Our findings reveal that fragranced body odor can affect judgments of self-esteem and attractiveness.”

*L. 459-476 Sorokowska et al 2016 also reported differences in conveyed impressions for natural vs. fragranced samples. Please discuss.*

Following the invitation to discuss this point, we have added the following sentences on this point (line 538-546):

“Similarly, previous work has also shown that using fragranced cosmetics can improve judgments of attractiveness and pleasantness based on body odors, and at the same time can partly obscure the perception of certain personality traits (Sorokowska, Sorokowski & Havlicek, 2016). In line with these and other studies (Demattè, Österbauer, & Spence, 2007; Gaby & Zayas, 2017; cf. Ferdenzi, Ortegón, Delplanque, Baldovini & Bensafi, 2020), the results suggest that just as people alter their voice, facial expression and face appearance to convey a particular impression to someone else (Kramer & Ward, 2010; Toma et al., 2008), fragrances are effectively employed to alter one’s chemical impression.”

*L. 476-490 - this paragraph is difficult to follow. Please rephrase or remove it.*

Following the suggestion of the reviewer, we have rephrased this paragraph and hopefully made its point much clearer (lines 547-568):

“Whereas previous studies on chemosignaling (e.g., De Groot et al., 2015; Pause, Ohrt, Prehn, & Ferstl, 2004) find mostly implicit effects of body odors, and no explicit perception effects, we found no implicit effects of odors, but only explicit perception effects of body odors. These perceivable differences in natural body odors did not affect the participants. In psychology, implicit measures are generally preferred as evidence for a specific effect, as they are believed to provide more meaningful information than explicit judgments (e.g., Eastwick, Eagly, Finkel, & Johnson, 2011). Indeed, smells do not always receive overt attention, with many people not noticing but the most extreme changes in their surrounding *smellscape* (Köster et al., 2014). At the same time, some people are much more aware of the smells in their surroundings. There are cultural differences in (social) odor awareness, but within country factors also play a large role (Ferdenzi, Coureaud, Camos & Schaal, 2008; Sorokowska et al., 2018). For example wine and coffee experts are more aware of their sense of smell (Croijmans & Majid, 2016), and perfumers, women, and hunter-gatherers (see for a review Majid, Speed, Croijmans, & Arshamian, 2017). This raises the question of what the effects of awareness on chemosignaling are. If one person barely takes notice of the strongest smells, while the next person is able to volitionally sniff out the subtlest differences in body odors (e.g., the smell of Parkinson’s disease; Trivedi et al., 2019), that raises questions whether there are individual differences in the effects of chemosignaling. An interesting alley for future studies would be to investigate whether particular social chemosignaling effects are only present when perceived unattentively (i.e., implicitly), or whether they lose, or--equally likely--gain, power when their presence is attentively noticed, for example by a highly aware and trained perfumer.”
