# Supplementary material for: The role of fragrance and self-esteem in perception of body odors and impressions of others
Source: PLoS One. 2021 Nov 15;16(11):e0258773. doi: 10.1371/journal.pone.0258773 (PMC8592444; doi:10.1371/journal.pone.0258773)
Supplement: Attachment — Submitted filename: Letter to reviewers R2 20210816.docx [file pone.0258773.s003.docx]

Dear Editor,

Thank you for the opportunity to improve our manuscript. Below, we respond on each point raised by the reviewer, with the reviewer’s comments in *italic* typeface, and our response in normal typeface.

*Introduction:
p. 6 l. 111: “– blocking sweat excretion from the underarm, and thus any –“ – there is a word missing here.*

We have changed this to (lines 109-113):

‘In similar vein, Dalton, Mauté, Jean and Wilson (2013) demonstrated that when participants were exposed to sweat from female participants who applied antiperspirant – blocking sweat excretion from the underarm– they rated the female participants as more confident, trustworthy, and competent in a stressful situation, compared to when they were exposed to their ‘natural’ sweat odor.’

*p. 6 l. 116-120: the reference “(Craig Roberts et al., 2009)” is not relevant and incorrectly formatted.*

We have removed this reference here.

*p. 6 l. 122-123: “the psychological reasons underlying fragrance use, and the effects of this, has not been extensively investigated.” – This statement is not true, as there is a large body of literature showing multiple connections between psychological factors and fragrance use. Please reword or remove this sentence altogether.*

We agree that this sentence was stated imprecisely, and have removed the second part of the sentence so that it reads (line 121-122):

“Taken together, fragrances appear to change the impression we have on others in different ways.”

*Methods:
p. 8 l. 161: please clarify that you aimed to obtain 32 samples and invited more male senders to account for potential dropouts. Thank you for presenting the process in Figure 1, it is very useful.*

We added a clarifying sentence in line 164:

“We initially aimed to obtain samples from 32 male participants, and invited more participants to account for potential dropouts.”

*p. 9 l. 193: “yielding n = 46 as group sizes” – group size per condition or a total sample size? Please verify and correct. The study is significantly underpowered if this is the required sample size per condition (I hope this is not the case).*

We would like to clarify that this study has a within-subjects design. Thus, n=46 is both the group size per condition as well as the total sample size. We have clarified this at this point in the text (line 196):

“These effect sizes were pooled (*d* = .35) and converted to eta^2^ (η^2^ = .0297), yielding *n* = 46 as group size sufficiently powered to find a within-subjects effect.”

*p. 9 l. 201-203: you wrote that “Perceivers were included based on their self-reported normal olfactory functioning, and completed a three Sniffin’ stick normosmia test to confirm normal sense of smell* *(Hummel, Pfetzing, & Lötsch, 2010).” To confirm normal sense of smell based on the q-stics you would need to obtain the score of 3 for each participant, which means that many of your perceivers could have been hyposmic or even anosmic (self-reports are not very reliable, as shown by several publications). Please discuss this problem as a limitation of your study.*

We agree that it is a possibility that some participants may have been hyposmic to some of the sniffin’ stick odors given their score on this test, and that potentially, this is a limitation to our findings. At the same time, several publications have also shown odor identification is very difficult (e.g., De Wijk, Schab & Cain, 2014; Yeshurun & Sobel, 2010). And we cannot be sure that these scores are predictive of the ability to smell body odour or fragrance. Therefore, we cannot be sure whether these scores indeed impacted the results. To address the reviewer’s concern, we have re-analysed the data, using the subset of participants that obtained a score of 3 correct identifications (n = 41), using our data available from the public repository (<https://osf.io/xwtnq/>). We have added this syntax code to the syntax file in the data repository.

This analysis yields results in line with the conclusions in our paper:

- For rated pleasantness, a significant main effect of fragrance use, *p* < .001, and for sender type, *p* =.002, but no interaction, *p* = .093.
- For rated intensity, a significant main effect for fragrance use, *p* < .001, and for sender type, *p* = .002, and an interaction between fragrance and sender type, *p* = .006.
- For rated familiarity, there was a main effect of fragrance use, *p* < .001, but no main effect for sender type, *p* = .175, nor was there a significant interaction, *p* = .133.
- For rated self-esteem, there was a main effect of fragrance use, *p* = .026, but no main effect for sender type, *p* = .216, nor was there a significant interaction, *p* = .625.
- For rated attractiveness, there was a main effect of fragrance use, *p* = .036, but no main effect for sender type, *p* = .242, nor was there a significant interaction, *p* = .606.
- For rated extraversion, there were no significant main effects or interactions.

We have included a discussion on this limitation in lines 573-585:

Another limitation is the use of the quick smell test (Hummel, Pfetzing, & Lötsch, 2010), instead of a full test battery for olfactory function, to assess whether our perceiver participants were not impaired in their sense of smell (i.e., hyposmic or anosmic). By not excluding participants that did not score three correct identifications, it is possible that some of the participants in our perceiver experiment were hyposmic or even anosmic, even though they did not report this themselves. One methodological explanation for the absence of an effect of self-esteem status of the senders on rated self-esteem could lie in the fact that perceiver participants were not sensitive enough to detect these subtle differences in sweat. To investigate this possibility, the main analyses were repeated, including only the participants that scored 3 out of 3 correct (n = 41; the syntax for this analysis is available on <https://osf.io/xwtnq/>). This analysis yielded the same results, suggesting that in our sample, hyposmia does not explain the absence of an effect of sender self-esteem status on perceiver behavior.

*Results
p.15-16 l. 354-359: please move this part to the Discussion.*

Following the reviewer’s suggestion, we have integrated this paragraph with the limitations section in the discussion (line 564-573):

The results suggest that female perceivers are able to discriminate between sweat from participants with high versus low self-esteem, but that this effect disappeared when the body odor was masked with fragrance. But interpreting these results should be done carefully, since sweat was pooled, every 8 of 64 stimuli contained sweat from the same individual, to mitigate individual differences in the sweat. However, particularly strong smelling sweat from one or a few individuals in one condition may have given a distinctive cue to many perceivers when discriminating the sweat, despite the efforts and hygiene standards applied in the sender protocol. Future studies with larger samples, collected using more ecological methods, may be able to overcome this limitation (cf. De Groot, Croijmans & Smeets, 2020).

*Table 2: Please modify this table to present the rated characteristics in consecutive rows, and columns low self-esteem donors (no deo-deo) followed by high self-esteem donors (no deo-deo) in columns. Perhaps you could also add a column “significant effects” and just briefly state, e.g., “Frag”, “Send”, “Frag*Send” This will help the reader grasp all your findings (I must admit I needed to read the whole section a few times to get the overall picture).*

We agree with the reviewer that this table needed a makeover. We have switched rows and columns, and added a column on what effects were found to be significant, on page 18, line 391.

*l. 409-448: Please write clearer that the psychological judgments refer to ratings of videos of men other than your senders.*
To make this more clear, we have added the following to the text (line 405-406):

To further investigate the effects of low and high self-esteem sweat on perceiver judgements of self-esteem, attractiveness, and extraversion, of videos showing male dating show contestants different to the sender participants, 2-way ANOVAs were done, again with Sender type and Fragrance as within participant factors (see Fig. 3).

We have also added the following statement to the notes for fig. 3 (page 20):

Perceiver participants rated videos that portrayed males (i.e., dating show contestants) who were not the sender participants (see the Psychological Judgments paragraph in the Methods section).

*Discussion
Please present the limitations of your study in a separate paragraph.*

We have added a paragraph on the limitations of our study in lines 560-585:

The present study was limited by a number of issues. Men and women differ in their ability to smell. Here, we have tested only female perceivers, smelling sweat from male senders. To be able to generalize to larger populations and to understand the underlying mechanisms of social chemosignaling, future research needs to include experimental groups including male perceivers, and female senders. The results suggest that female perceivers are able to discriminate between sweat from participants with high versus low self-esteem, but that this effect disappeared when the body odor was masked with fragrance. But interpreting these results should be done carefully, since sweat was pooled, every 8 of 64 stimuli contained sweat from the same individual, to mitigate individual differences in the sweat. However, particularly strong smelling sweat from one or a few individuals in one condition may have given a distinctive cue to many perceivers when discriminating the sweat, despite the efforts and hygiene standards applied in the sender protocol. Future studies with larger samples, collected using more ecological methods, may be able to overcome this limitation (cf. De Groot, Croijmans & Smeets, 2020). Another limitation is the use of the quick smell test (Hummel, Pfetzing, & Lötsch, 2010), instead of a full test battery for olfactory function, to assess whether our perceiver participants were not impaired in their sense of smell (i.e., hyposmic or anosmic). By not excluding participants that did not score three correct identifications, it is possible that some of the participants in our perceiver experiment were hyposmic or even anosmic, even though they did not report this themselves. One methodological explanation for the absence of an effect of self-esteem status of the senders on rated self-esteem could lie in the fact that perceiver participants were not sensitive enough to detect these subtle differences in sweat. To investigate this possibility, the main analyses were repeated, including only the participants that scored 3 out of 3 correct (n = 41; the syntax for this analysis is available on <https://osf.io/xwtnq/>). This analysis yielded the same results, suggesting that in our sample, hyposmia does not explain the absence of an effect of sender self-esteem status on perceiver behavior.

**References**

de Wijk, R. A., Schab, F. R., & Cain, W. S. (2014). Odor identification. In *Memory for odors* (pp. 29-46). Psychology Press.

Yeshurun, Y., & Sobel, N. (2010). An odor is not worth a thousand words: from multidimensional odors to unidimensional odor objects. *Annual review of psychology*, *61*, 219-241.
